# Milk Intolerance, Beta-Casein and Lactose

**DOI:** 10.3390/nu7095339

**Published:** 2015-08-31

**Authors:** Sebely Pal, Keith Woodford, Sonja Kukuljan, Suleen Ho

**Affiliations:** 1 School of Public Health, Curtin Health Innovation Research Institute, Curtin University, GPO Box U1987, Perth WA 6845, Australia; E-Mail: Suleen.Ho@curtin.edu.au; 2 Agricultural Management Group, Lincoln University, PO Box 85084, Lincoln 7647, Christchurch, New Zealand; E-Mail: keith.woodford@lincoln.ac.nz; 3 The a2 Milk Company (Australia) Pty Ltd, PO Box 180, Kew East, Victoria 3102, Australia; E-Mail: sonja.kukuljan@a2Milk.com

**Keywords:** milk consumption, lactose, beta-casein, lactose intolerance

## Abstract

True lactose intolerance (symptoms stemming from lactose malabsorption) is less common than is widely perceived, and should be viewed as just one potential cause of cows’ milk intolerance. There is increasing evidence that A1 beta-casein, a protein produced by a major proportion of European-origin cattle but not purebred Asian or African cattle, is also associated with cows’ milk intolerance. In humans, digestion of bovine A1 beta-casein, but not the alternative A2 beta-casein, releases beta-casomorphin-7, which activates μ-opioid receptors expressed throughout the gastrointestinal tract and body. Studies in rodents show that milk containing A1 beta-casein significantly increases gastrointestinal transit time, production of dipeptidyl peptidase-4 and the inflammatory marker myeloperoxidase compared with milk containing A2 beta-casein. Co-administration of the opioid receptor antagonist naloxone blocks the myeloperoxidase and gastrointestinal motility effects, indicating opioid signaling pathway involvement. In humans, a double-blind, randomized cross-over study showed that participants consuming A1 beta-casein type cows’ milk experienced statistically significantly higher Bristol stool values compared with those receiving A2 beta-casein milk. Additionally, a statistically significant positive association between abdominal pain and stool consistency was observed when participants consumed the A1 but not the A2 diet. Further studies of the role of A1 beta-casein in milk intolerance are needed.

## 1. Introduction

There is a widespread assumption within both general society and among healthcare professionals that the dominant cause of milk intolerance is insufficient lactase enzyme activity. However, the evidence, as summarized in the 2010 National Institutes of Health consensus statement on lactose intolerance and health, is that “many who self-report lactose intolerance show no evidence of lactose malabsorption. Thus, the cause of their gastrointestinal symptoms is unlikely to be related to lactose” [1]. Providing an alternative mechanism, there is now an increasing body of evidence that bovine beta-casomorphin-7 (BCM-7), derived from A1 beta-casein, is also an important contributor to milk intolerance syndrome. It is that evidence that we discuss here, including the potential for interactions between lactose intolerance and A1 beta-casein intolerance.

## 2. Literature Search and Selection of Studies for Review

The objective of this review was to assess the evidence that bovine BCM-7, which is derived from A1 beta-casein, contributes to milk intolerance syndrome.

*In vitro* and *in vivo* animal studies and human clinical studies reporting outcomes relevant to the formation of BCM-7 or other beta-casomorphins in the gastrointestinal system, or other outcomes relevant to the formation of these peptides, were included in this review. Studies involving milk, milk products and beta-casein were also considered. For *in vivo* animal and human clinical studies, only studies that assessed outcomes following oral administration were included. Relevant outcome measures included the release of beta-casomorphins in actual or simulated gastrointestinal digestion of milk, milk products or beta-casein; opioid agonist activity following digestion of milk, milk products or beta-casein including differences in gastrointestinal transit time; and variations in other biomarkers relevant to the gastrointestinal system following consumption of milk, milk products or beta-casein.

Literature searches were undertaken using Medline/PubMed on 20 October 2014 using the following search terms: Casomorphin; Beta-casomorphin; Beta-casomorphin-7; Beta-casomorphine; Beta-casomorphine-7; A1_beta casein OR A2_beta casein; b-cm 7 OR bcm7 OR bcm-7; beta-casein AND A1 OR A2; and A2 AND Milk. The authors’ existing EndNote X5 reference management software library was also used to identify any additional papers not captured by the literature searches. Studies published since October 2014 were added manually. Data were extracted manually. Studies were assessed manually for bias, based on the information provided in each publication. We focused on studies relevant to the stated aim of the current review.

## 3. Beta-Caseins and BCM-7

**Figure 1:**
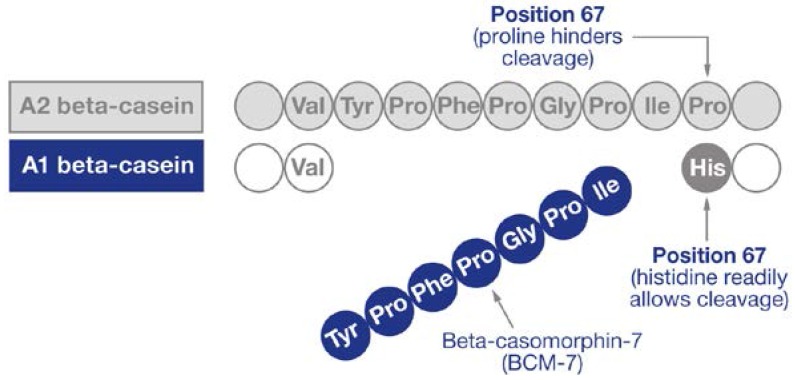
Release of beta-casomorphin-7. Adapted from Woodford [12] (reproduced with permission of the publisher).

Beta-casein proteins make up approximately 30% of the total protein of cows’ milk [2] and may be present as one of two major genetic variants: A1 and A2 [3]. A2 beta-casein is recognized as the original beta-casein variant because it existed before a proline^67^ to histidine^67^ point mutation caused the appearance of A1 beta-casein in some European herds some 5000–10,000 years ago [4]. Once milk or milk products are consumed, the action of digestive enzymes in the gut on A1 beta-casein releases the bioactive opioid peptide BCM-7 [5,6,7,8,9]. In contrast, A2 beta-casein releases much less and probably minimal amounts of BCM-7 under normal gut conditions (Figure 1) [10]. However, it is notable that under specific *in vitro* conditions relating to pH and enzyme combinations not found in the human gut, A2 beta-casein can also release some BCM-7 [11].

The two major A1 and A2 beta-casein variants can be considered as beta-casein “families”, which include at least 10 sub-variants. Those within the A1 family are B, C, D, F and G. Those within the A2 family are A3, E, H1, H2 and I [13]. Whether the different tertiary structures of the sub-variants within the A1 family have any effect on the release of BCM-7 is unproven. However, there is some evidence that the B sub-variant may result in a particularly high release of BCM-7 [7].

A1 beta-casein has only been found in cattle of European origin. Purebred Asian and African cattle produce milk containing only the A2 beta-casein type, although some cattle presenting phenotypically as Asian or African cattle may produce A1 beta-casein as a consequence of crossbred ancestry. The relative prevalence of A1 and A2 beta-casein in cattle is breed-dependent, with Northern European breeds generally having higher levels of A1 beta-casein than Southern European breeds. Guernsey and Fleckvieh breeds are generally considered to have a particularly high A2 allele frequency. However, within any specific herd, basing the estimation of allele frequency on breed category is not reliable. In the herds in many Western countries, the ratio of A1:A2 is approximately 1:1 [10]. Herd testing for beta-casein alleles can be undertaken using DNA analysis, which is available commercially in some countries. Converting a specific herd by selective breeding to eliminate all A1 beta-casein from the milk can be achieved within 4 years using intensive methods of animal selection that incorporate the use of sex-selected semen, but more typically this will take 5–8 years or longer [14].

The *in vivo* release of BCM-7 from each liter of bovine milk will depend on the protein content of the milk (which is in turn affected by the breed, animal feeding and component standardization procedures during milk processing), the proportion of A1 and A2 beta-casein, and possibly the specific gastrointestinal conditions of the individual. There is now clear evidence that BCM-7 is released not only from milk but also from yoghurt and cheese, and in all likelihood any milk product [6,7]. There is also evidence that there is modest release of BCM-7 in the cheese- and yoghurt-making processes, but that during the latter, certain bacteria present in yoghurt may hydrolyze BCM-7 [15,16]. Whether such bacteria consumed in yoghurt also have a similar influence within the human gastrointestinal tract is unknown.

In human milk, beta-casein is of the A2 type, with a proline at the equivalent position on the beta-casein protein chain [17]. Human BCM-7 has a different amino acid sequence to bovine BCM-7, with homology in five of seven amino acids (differing amino acids at positions four and five) [17,18], and considerably weaker opioid activity [19,20]. Wada and Lonnerdal [18] examined non-digested and *in vitro*-digested human milk, and reported the presence of human BCM-9 (which has a proline at position eight), but not human BCM-7 or BCM-5 (*i.e.*, BCM-5 is the truncated form of BCM-7). However, Jarmolowska *et al.* reported the presence of both human BCM-5 and BCM-7 in colostrum (averaging 5 and 3 μg/mL respectively), but at 2 months into the lactation period, the authors reported much lower quantities [21]. It has been postulated that casomorphin functionality in neonates may relate to maternal bonding, gastrointestinal function, mucosal development and sleep induction [21].

## 4. Opioid Characteristics of Casomorphins

Beta-casomorphins are μ-opioid receptor ligands [8,22,23,24,25]. The natural casomorphins of relevance are BCM-5, BCM-7 and BCM-9. The most potent of these natural opioids is BCM-5. In theory, BCM-5 could be released from BCM-7 within the human biological system by the human equivalent of the enzyme carboxypeptidase Y [23], and there is some evidence supporting this [7,26]. Wasilewska *et al.* reported that bovine BCM-5 is present in the serum of exclusively breastfed human babies whose mothers consumed bovine milk [26]. However, it has not been demonstrated whether the BCM-5 was hydrolyzed from BCM-7 by the mothers and passed via their breastmilk to the infants, or whether the mothers passed bovine BCM-7 via their breastmilk to the infants where it was subsequently degraded to BCM-5 before or after intestinal absorption.

The second most potent natural casomorphin is BCM-7 and it is the major focus of this review. Bovine BCM-7 has been identified in human jejunal contents following milk-protein feeding at levels consistent with pharmacological effects, with 4 mg BCM-7 released from 30 g of casein after 2 h of digestion, with further release thereafter [5]. It has also been identified in the blood of human infants [27,28] and urine of children [29].

The third natural casomorphin of interest is BCM-9. This peptide is released from the A2 type variant of beta-casein [5,18], but it is unlikely to be a peptide of importance in relation to A1 beta-casein because of the histidine at position 67 on A1 beta-casein. In this regard, it is notable that Boutrou *et al.* [5] report in their supplementary data considerable quantities of BCM-9 with a proline at position 67 (therefore, by definition, derived from A2 beta-casein), whereas there was almost no BCM-9 with a histidine at this position (*i.e.*, from A1 beta-casein). This provides supporting evidence that BCM-9 with a histidine at position 67 is readily broken down at the histidine cleavage point to BCM-7 within the gastrointestinal system, whereas BCM-9 with a proline at position 67 is cleavage resistant.

BCM-9 does exhibit opioid properties, but with a binding affinity to μ-opioid receptors approximately one quarter that of BCM-7 [8]. These findings are consistent with those of Barnett *et al.* who conducted a study in rats [30]. They found that, while A1 beta-casein exhibited a range of gastrointestinal effects that were blocked by the μ-opioid receptor antagonist naloxone, no such effects occurred with A2 beta-casein following naloxone administration [30]. Of interest, BCM-9 has been identified as having antihypertensive properties [31].

## 5. Beta-Caseins, Beta-Casomorphins and Delayed Intestinal Transit

μ-Opioid receptors are expressed widely in humans, including in the gastrointestinal tract [32]. μ-Opioid receptor activation is known to affect the mechanics of intestinal propulsion [33] and to play an important role physiologically in controlling gastrointestinal function, including regulating motility, mucus production and hormone production [34]. Gastrointestinal μ-opioid receptor activation occurs on both enteric neurons and directly on epithelial cells [35,36]. μ-Opioid receptor agonists are known to delay gastrointestinal transit time in humans, in a naloxone-reversible manner. For example, the opioid codeine has been shown in humans to significantly delay small intestinal and consequently overall colonic transit time [37]. Additionally, in humans consuming high-fiber diets, Stephen *et al.* showed that administration of a sufficient codeine dose to double gastrointestinal transit time results in a major controlling influence over the colonic microflora, and thereby colonic function, with codeine significantly decreasing both total stool mass and bacterial mass [38]. They concluded that “differences in bowel habit and microbial cell metabolism between individuals on similar diets are largely attributable to differences in mean transit time” [38]. This study also demonstrated that the quantity of codeine needed to double transit time varied considerably between individuals, which reinforces the importance of population sub-groups when considering intolerance issues.

Several other studies provide direct evidence that casein and/or its derivatives decrease gastrointestinal motility, in part by reducing the frequency and amplitude of intestinal contractions [39,40,41,42,43]. In the canine small intestine, a comparison of casein and soy protein on various small intestinal motility measures (e.g., force and contraction frequency) showed that casein reduced these parameters significantly and that pretreatment with naloxone blocked this effect [40], suggesting a role for exogenous opioids. Similarly, casein was also shown to delay gastrointestinal transit time in rats compared with whey protein, with naloxone partially or completely reversing these casein effects, again indicating that the opioid activity of casein delays transit time [43]. In rat pups fed either intact casein powder or extensively hydrolyzed casein, small intestinal transit time was delayed [41], and the effect of the intact casein on delaying transit time was prevented with naloxone administration. These results suggest that peptides with opioid activity are released during digestion of intact casein, which can cause gastrointestinal transit time delays. This effect was not evident following rat pup feeding with extensively hydrolyzed casein.

A recent animal study investigating the effects of A1 *versus* A2 beta-casein on gastrointestinal transit has shown that A1 beta-casein delays gastrointestinal transit time relative to A2 beta-casein feeding [30]. Using Wistar rats fed A1 or A2 beta-casein type milk-based diets for 36 or 84 h, Barnett *et al.* showed that the A1 beta-casein diets delayed gastrointestinal transit time compared with the A2 beta-casein diets [30]. Co-administration of naloxone blocked the effects of the A1 diet on transit time, but had no effects in rats fed the A2 diet. The results indicate that the A1 diet has direct effects on gastrointestinal function by slowing transit time, and provides further support for a role for opioid signaling pathways in the effects of A1 beta-casein.

## 6. Inflammatory and Immune Responses to Casomorphins in the Gastrointestinal System

There is wide-ranging evidence for both inflammatory and immune responses to casomorphins within the gastrointestinal system. However, the overall implications of these responses are not fully understood. It has been shown in both rats [30] and mice [44] that A1 beta-casein is associated with increased levels of the inflammatory marker myeloperoxidase (MPO) in the colon. This effect is eliminated by administration of naloxone, indicating that it is an opioid-dependent response. Interestingly, intestinal inflammation enhances the potency of μ-opioid receptor agonists in inhibiting gastrointestinal transit, and increases the expression of μ-opioid receptors in the mouse intestine [45]. It has also been shown in rats that A1 beta-casein stimulates the production of the enzyme dipeptidyl peptidase 4 (DPP4) in the jejunum [30]. However, this effect is not attenuated by naloxone administration, indicating a non-opioid effect of A1 beta-casein on DPP4. The full implications of this are not understood, but it is notable that DPP4 degrades the gut incretin hormones rapidly [46]. In humans, incretin hormones modulate insulin and glucose metabolism [47] and affect antroduodenal motility [48]. DPP4 inhibitors are now widely used in the management of type 2 diabetes mellitus.

BCM-7 is also known to increase mucin production within the gastrointestinal system via an opioid pathway [34,49]. Gastrointestinal mucus provides a protective barrier between the epithelium and the lumen; however, excessive production has the potential to disrupt gastrointestinal function and interfere with commensal bacteria. It has been shown in two *in vitro* studies that BCM-7 alters lymphocyte proliferation, also via an opioid-dependent pathway [50,51]. The full physiological relevance of the immunomodulatory effects of BCM-7 in animals and humans requires further investigation.

More recently, Ul Haq *et al.* examined possible mechanisms underlying previously observed proinflammatory effects of BCM-7 [52]. In this study, mice were administered BCM-7 or BCM-5 orally, and both peptides resulted in increased expression of inflammatory markers (MPO, monocyte chemotactic protein-1 and interleukin-4). Increased levels of immunoglobulins, enhanced leukocyte infiltration into intestinal villi, and increased expression of Toll-like receptors in the gut were also observed. The authors concluded that both peptides stimulate inflammatory responses through the Th_2_ pathway. The same research group reported similar gastrointestinal immune effects in mice fed a milk-free basal diet supplemented with A1 relative to mice fed a diet supplemented with A2 beta-casein [44]. The diet containing A1 beta-casein had proinflammatory effects in the gut (increased levels of inflammatory markers and immunoglobulins, leukocyte infiltration and Toll-like receptor expression). These effects were not observed in mice fed A2 beta-casein. Taken together, these results highlight the potential proinflammatory effects of A1 beta-casein, and suggest pathways by which A1 beta-casein might contribute to a variety of clinical conditions, including gastrointestinal disorders.

## 7. Clinical Studies of Beta-Casein Effects in the Gastrointestinal System

Much of the human evidence for intolerance to A1 *versus* A2 beta-casein is observational and anecdotal, and has the potential to be influenced by the lack of a controlled environment. However, there are two clinical studies of relevance. The first, undertaken in Newcastle, Australia, aimed to investigate the effect of A1 and A2 beta-caseins on constipation in young children who suffered chronically from this condition [53]. The rationale for the trial was the considerable literature linking childhood constipation with milk, but with the causative factor being unresolved [54,55]. The authors reported 81% resolution of constipation during the milk-free washout period, 79% resolution during the A2 epoch and 57% resolution with A1 beta-casein [53]. However, with only 21 children completing the trial, the results were not statistically significant. Accordingly, the results were reported as showing no difference between treatments, although an alternative interpretation would have been that the trial, despite showing results of potential clinical importance, lacked sufficient statistical power and that further studies are needed. It is also notable that both the A2 and the A1 milk were commercially sourced, and that the beta-casein proportions were not analyzed or standardized. The A1 treatment was standard commercial milk, and although this is sometimes referred to as “A1 milk”, at that time in Australia it would have typically contained A1 and A2 beta-casein in approximately equal proportions. The beta-casein composition of the “A2 milk” used in this trial is also unknown, as the so-called “A2 milk” was sourced from a private Jersey dairy farm, and was therefore unlikely to be free of A1 beta-casein. Furthermore, the milk treatments used in the study comprised 400 mL/day, which was apparently lower than pre-treatment consumption for many of the participants. It is therefore possible that this low consumption may have contributed to the resolution of constipation levels independent of a particular treatment.

The second clinical trial comparing the gastrointestinal effects of A1 *versus* A2 beta-casein was conducted at Curtin University, Western Australia [56]. This trial comprised 36 participants at study completion. Although it was initially planned to recruit those with perceived milk intolerance, all participants had to be willing to drink 750 mL of milk per day. This led to the self-exclusion of many potential participants who had perceived milk intolerance. Accordingly, only eight of the 36 who completed the study considered themselves milk intolerant *ex ante*. The trial had a blinded cross-over design. Either A1 or A2 milk was consumed for a period of 2 weeks, followed by a 2-week washout period. Participants then crossed over to the second treatment. Key outcomes were statistically different Bristol Stool Scale measures (A1 milk, 3.87 *versus* A2 milk, 3.56, *p* = 0.04) with higher values (*i.e.*, looser and more runny stools) on A1 than A2 milk. These differences remained significant when participants reporting milk intolerance prior to the trial were excluded (thus, there were differences in stool outputs in people whom reported themselves to be milk tolerant). Particularly notable was a strong relationship between abdominal pain and increased stool looseness across all participants on the A1 diet (*r* = 0.520, *p* = 0.001), but not on the A2 diet (*r* = −0.13, *p* = 0.43). The difference between these two correlations (0.52 *versus* −0.13) was highly significant (*p* < 0.001). Similarly, while receiving A1 milk, higher gut inflammation (fecal calprotectin) correlated with higher abdominal pain (*r* = 0.46, *p* = 0.005) and higher bloating (*r* = 0.36, *p* = 0.03) scores. These relationships were absent in the same people when they received A2 milk. Again, the difference in the correlation measures was significant for: (i) gut inflammation and abdominal pain (A1, 0.46 *vs.* A2, 0.03; *p* = 0.02); and (ii) gut inflammation and bloating (A1, 0.36 *vs.* A2, −0.02; *p* = 0.05).

In contrast, differences in subjective measures of intolerance were not statistically significant. However, there were treatment differences in subjective measures of intolerance amongst the eight participants who considered themselves milk intolerant, which are of potential clinical significance.

The interpretation of these clinical results requires an appreciation of the body of evidence previously discussed in this review. The expected effect of BCM-7 on gastrointestinal transit is to disrupt the propagation of peristaltic contractions, and when considered in isolation, it might seem reasonable to assume that this would present as constipation. However, the significantly higher Bristol Stool Scale values in participants receiving A1 compared with A2 beta-casein diets may instead be caused by a combination of gastrointestinal transit delay and proinflammatory factors, with transit delays potentially providing additional opportunity for fermentable oligosaccharides to undergo gas-forming and stool-softening degradation. Prior evidence showing that intestinal inflammation is associated with malabsorption of fluids, nutrients and electrolytes [57,58] supports this proposition. This explanation is also consistent with the significant and positive association between abdominal pain and stool consistency on the A1 diet [56]. Accordingly, it is reasonable to hypothesize that the delayed transit effects of BCM-7 may lead to looser stools together with proinflammatory effects in at least some people. The results of Ho *et al.* [56] are consistent with this interpretation.

It is also well understood that milk intolerances and gastrointestinal sensitivities will exhibit differently in different individuals. Accordingly, and given the multiplicity of biological effects, it is not unreasonable to expect that in some people, the dominant symptom from BCM-7 may be constipation, whereas in others it might be an increased looseness of stools. The close association in the A1 epoch between looser stools and measures of subjective discomfort, and the association between higher fecal calprotectin values and subjective intolerance measures when participants were receiving the A1 diet in the Ho *et al.* study [56] is also supportive of the hypothesis that delayed gastrointestinal transit with associated discomfort followed by loose stools may be expected in at least some individuals.

## 8. The Potential for BCM-7 and Lactose Interactions

The likelihood of BCM-7 and lactose interactions deserves consideration. There are various mechanisms by which this might occur. The first is that the inflammatory characteristics of BCM-7 may affect lactase production/activity and possibly exacerbate existing hypolactasia and consequent lactose malabsorption symptoms in susceptible individuals. The second is that colonic inflammation affects the processing of malabsorbed lactose, possibly via changes in the gut microbiota that occur with gut inflammation [59]. The third is that the delayed gastrointestinal transit leads to increased opportunity for lactose fermentation (and the opportunity for fermentation of other dietary-derived oligosaccharides). These possibilities are consistent with the current state of knowledge of gastrointestinal symptoms related to lactose malabsorption. However, all need to be tested in clinical investigations.

## 9. Conclusions

Milk intolerance is a complex problem of importance both to public health and individual health. It is clear that lactose malabsorption (and consequent symptoms) is one element of the syndrome, but it is also evident that there are other factors at play. The potential role of A1 beta-casein is arguably the prime candidate requiring closer scrutiny if understanding is to be advanced.

It is important to note the considerable advancements relating to A1 beta-casein and BCM-7 that have been made since the European Food Safety Authority (2009) report on the possible health effects of beta-casomorphins and BCM-7 [10]. At that time, the EFSA recognized that BCM-7 exerts biological activities such as regulatory effects on gastrointestinal motility and on gastric and pancreatic secretions. However, they concluded that a “cause and effect” relationship could not be established between the dietary intake of BCM-7 and assessed non-communicable diseases, which included type 1 diabetes, heart disease and autistic spectrum disorders. Their conclusion was reached partly because BCM-7 had not been detected in human blood following milk or casein intake, and partly because there was insufficient knowledge about the levels of BCM-7 likely to originate from the digestion of milk and its products. Additionally, the EFSA report did not specifically address intolerance issues.

Several conclusive studies have been published since the EFSA (2009) report (as discussed in the current review), which report that the opioid peptide BCM-7 is released in pharmacologically relevant quantities from digestion of A1 beta-casein, but not from A2 beta-casein in the human gastrointestinal system. It is also clear that BCM-7 has a range of effects within both *in vitro* models and *in vivo* in animal experiments. These effects include those on gastrointestinal motility, proinflammatory and immunomodulatory outcomes. Most, but not necessarily all of these effects are opioid-related. Given the complexity of the relationships, it is reasonable to expect that exhibited symptoms will vary between individuals.

Data from human clinical trials are limited, but statistically significant results from the recent study by Ho *et al.* are consistent with prior knowledge and scientific hypotheses drawn from *in vitro* investigations and animal trials [56]. It is notable that in this study, significant differences in stool consistency were identified in a cohort of people who had no prior awareness of milk intolerance [56], which may be caused by proinflammatory factors alongside effects on gastrointestinal transit time [30,44,56]. Further studies are required to confirm these observations.

Given the specificity of A1 beta-casein to cattle of European origin, and hence also the release of BCM-7, the current evidence also provides a contributory explanation as to why some people report anecdotally that they can tolerate milk from mammals such as sheep [60] and goats (GenBank Accession No. AJ011019.3) (which contain A2-like beta-casein and not A1, because they have a proline at the homologous position on their beta-casein chains), but not cows. It is also clear that it is feasible for dairy farmers to breed herds of bovine cows that are free of A1 beta-casein. Indeed such herds already exist and, where available, the dairy products are supported by consumers.
